# An association between BPDE-like DNA adduct levels and *CYP1A1* and *GSTM1* polymorphisma in pterygium

**Published:** 2010-04-08

**Authors:** Jai-Nien Tung, Heng-Hsiung Wu, Chun-Chi Chiang, Yi-Yu Tsai, Ming-Chih Chou, Huei Lee, Ya-Wen Cheng

**Affiliations:** 1 Institute of Medicine, Chung Shan Medical University, Taichung, Taiwan; 2 Department of Neurosurgery, Tungs’ Taichung MetroHarbor Hospital, Taichung, Taiwan; 3 Department of Ophthalmology, China Medical University Hospital, Taichung, Taiwan; 4 Institute of Medical & Molecular Toxicology, Chung Shan Medical University, Taichung, Taiwan; 5 Department of Medical Research, Chung Shan Medical University Hospital, Taichung, Taiwan

## Abstract

**Purpose:**

Benzo[a]pyrene 7,8-diol 9,10-epoxide (BPDE), an ultimate metabolite of benzo[a]pyrene, attacks deoxyguanosine to form a BPDE-N2-dG adduct resulting in *p53* mutations. Both cytochrome P4501A1 (CYP1A1) and glutathione S-transferase M1 (GSTM1) have been demonstrated to be involved in the metabolism of polycyclic aromatic hydrocarbons. The relationship between BPDE-like DNA adduct levels and *CYP1A1* and *GSTM1* gene polymorphisms in pterygium is not clear. Therefore, BPDE-like DNA adducts and *CYP1A1* and *GSTM1* polymorphisms were detected in this study to provide more molecular evidence to understand the cause of BPDE-like DNA adduct formation in pterygium.

**Methods:**

In this study, immunohistochemical staining using a polyclonal antibody on BPDE-like DNA adducts was performed on 103 pterygial specimens. For the analysis of *CYP1A1* and *GSTM1* polymorphisms, DNA samples were extracted from epithelial cells and then subjected to restriction fragment length polymorphism (RFLP) and polymerase chain reaction (PCR) for the determination of mutation and genotype of *CYP1A1* and *GSTM1*.

**Results:**

BPDE-like DNA adducts were detected in 33.0% (34/103) of the pterygium samples. The differences in DNA adduct levels were associated with the genetic polymorphisms of *CYP1A1* but not *GSTM1*. Additionally, the risk of BPDE-like DNA adduct formation for patients with *CYP1A1* m1/m2 (C/T) and**m2/m2 (T/T) was 9.675 fold higher than that of patients with *CYP1A1* m1/m1 (C/C) types (p=0.001, 95% Confidence Interval 2.451–38.185).

**Conclusions:**

Our data provide evidence that the BPDE-like DNA adduct formation in pterygium samples was associated with *CYP1A1* polymorphisms.

## Introduction

Pterygium is a chronic condition characterized by the encroachment of a fleshy triangle of conjunctival tissue into the cornea. It has long been considered a chronic degenerative condition; however, after finding abnormal expression of the p53 protein in epithelium, pterygium is now considered to be an ultraviolet-related uncontrolled cell proliferation, like a tumor [1-7]. The *p53* tumor suppressor gene is one of the most commonly mutated genes observed in human tumors. Mutations within the *p53* gene were detected in 15.7% of the pterygial samples of our previous study, and deletion mutations were found in the same samples with p53-negative staining, while substitution mutations were found in samples with p53-positive staining [8]. However, the cause of *p53* mutation in pterygium is still unclear. Polycyclic aromatic hydrocarbons (PAHs) might be responsible for the mutagenicity of airborne particulates in Taiwan [9,10]. The environmental pollutant, benzo[a]pyrene (BaP), which is one of the PAHs, has been found to cause *p53* mutations and then lung tumorigenesis. The levels of PAHs in airborne particulates in Taiwan are higher than levels found in other countries, especially levels of BaP, benzo[b]fluoranthrene, and benzo[g,h,i]perylene [9,10]. BaP 7,8-diol 9,10-epoxide (BPDE), an ultimate metabolite of BaP, attacks deoxyguanosine to form a BPDE-N2-dG adduct that results in *p53* mutations. The mutation hotspots of *p53* in human lung tumors (codons 154, 157, 158, 245, 248, and 273) are caused by the BPDE-N2-dG adduct [11]. Thus, an evaluation of DNA adducts induced by BaP and other PAHs is suitable as a risk marker for p53 mutation.

Bap is oxidized by a series of well-characterized enzymes, such as cytochrome p450 1A1, 2C9, and 3A4 [12,13]. A thymine/cytosine point mutation in the MspI restriction site of cytochrome P4501A1 (*CYP1A1*) has been reported to result in increased enzyme activity [14]. The *CYP1A1* MspI polymorphism has been linked to the susceptibility for smoking-related cancers, such as lung [15,16], colon, breast, and oral cancers [17]. Not only cytochrome P450 but also other enzymes, such as glutathion s-transferase M1 (GSTM1), was shown to be involved in BaP metabolism [18-20]. *GSTM1* has also been shown to be polymorphic. A deletion is responsible for the existence of a null allele associated with the lack of expression of a functional protein [21,22]. The polymorphic *GSTM1* null genotype has been found in 20–50% of populations of various ethnic origins, and this genotype has been correlated with the risk for various tobacco-related cancers [23-26]. Therefore, genetic polymorphisms of *CYP1A1* and *GSTM1* may contribute to BPDE-like DNA adduct formation and pterygium progression.

In this study, we try to detect the BPDE-like DNA adducts, using immunohistochemistry in 103 pterygium specimens, and we compare them with *CYP1A1* and *GSTM1* polymorphisms to understand the relationship between environmental exposure and genetic polymorphism in pterygium.

## Methods

### Patients and methods

Pterygial samples were harvested from 103 patients (68 males and 35 females) with primary pterygium undergoing pterygium surgery at China Medical University Hospital, Taichung, Taiwan. The age range was 52 to 85, and the average age was 70.2-years old. All specimens were formalin fixed and paraffin embedded. Then, 3-µm-thick sections were cut, mounted on glass, and dried overnight at 37 °C for immunohistochemical analysis. All participants were asked to submit a written informed consent approved by the Institutional Review Board of the Chung-Shan Medical University Hospital.

### Immunohistochemical analysis of BaP 7,8-diol 9,10-epoxide (BPDE)-like DNA adduct detection

All sections were deparaffinized in xylene, rehydrated with alcohol, and washed in PBS (3.2 mM Na_2_HPO_4_, 0.5 mM KH_2_PO_4_, 1.3 mM KCl, 135 mM NaCl, pH 7.4). This buffer was used for all subsequent washes. Sections for BPDE-like DNA adduct detection were heated in a microwave oven (TMO-2050; TATUNG, Taiwan) with 700 W power, twice, each time for 5 min in citrate buffer (pH 6.0). Anti-BPDE-like DNA adduct polyclonal antibody (which was kindly provided by Dr. Huei Lee, Institute of Medical & Molecular Toxicology, Chung Shan Medical University, Taichung, Taiwan; at a dilution of 1:1,000 [27]) was used as the primary antibody, and the incubation time was 60 min at room temperature (25 °C) followed by a conventional streptavidin peroxidase method for antibody detection  (LSAB Kit K675; DAKO,Glostrup, Denmark). The sections were incubated with biotinylated secondary antibody 10 min at room temperature (25 °C). After washed with PBS, the section were incubated with HRP conjugates Streptavidin 10 min at room temperature (25 °C). Signals were developed with 3, 3′-diaminobenzidine (LSAB Kit K675; DAKO) for 5 min and counter-stained with hematoxylin (DAKO). The positive and negative controls used for the BPDE immunohistochemical stain were lung tissue, [28] which had high and nondetectable BPDE DNA adduct levels, respectively, as analyzed by ^32^P-post labeling and enzyme-linked immunosorbent assay (ELISA) [28]. The results were evaluated independently by three observers and scored for the percentage of positive nuclei: score 0, no positive staining; score +, from 1% to 10%; score ++, from 11% to 50%; and score +++, more than 50% positive cells. In this study, scores of +, ++, and +++ were considered to be positive immunostaining, and a score of 0 was considered to be negative immunostaining.

### Polymorphisms of *CYP1A1* and *GSTM1*

DNA was extracted from the paraffin-embedded pterygium tissues for genetic polymorphism analysis [29]. DNA lysis buffer (10 mM Tris-HCl, pH 8.0, 0.1 M NaCl, 25 mM EDTA, and 0.5% SDS) was applied to lyse the epithelial cells on the slide, and then the DNA solution was transferred into an Eppendorf tube for traditional proteinase K digestion and phenol-chloroform extraction. The suspension was incubated at 56 °C for 2 h in the presence of proteinase K. The suspension was sequentially extracted with phenol-chloroform (25: 24).. Finally, the DNA was precipitated with 500 μl of 100% ethanol with an addition of linear polyacrylamide to increase DNA amounts [30]. Genotyping of the MspI polymorphism of *CYP1A1* was performed by PCR amplification using the primer set of 5′-TAG GAG TCT TGT CTC AGC CT-3′ and 5′-CAG TGA AGA GGT GTA GCC GCT-3′ [31]. The amplified products were digested with MspI and analyzed by electrophoresis on a 1.5% agarose gel. The MspI restriction site polymorphism resulted in three genotypes: a predominant homozygous m1 allele without the MspI site (genotype m1/m1; C/C), the heterozygote (genotype m1/m2; C/T) and a rare homozygous m2 allele with the MspI site (genotype m2/m2; T/T). Detailed information of the PCR assays can be found elsewhere [32]. Briefly, the PCR reaction containined: DNA 1 μl, 0.5 mM dNTP, 5 μl 10× reaction buffer, 2.5 U Taq polymorase and 0.5 mM primer. An initial denaturing step of 5 min at 94 °C followed by 35 cycles of 94 °C for 30 s, 60 °C for 45 s, and 72 °C for 1 min and then a final extension at 72 °C for six min. Genotypes of *GSTM1* were determined by the presence or absence of the PCR product, according to the method of Groppi et al. [32]. The genotypes of *GSTM1* are defined as present and null types. Two primers, 5′-GAA GGT GGC CTC CT-CC TTG G-3′ and 5′-AAT TCT GGA TTG TAG CAG AT-3′, were used for PCR. If samples had no PCR product, the PCR experiment was repeated with the addition of a set of β-actin (*ACTB*) primers together with *GSTM1* primers to confirm that the absence of the *GSTM1* PCR product represented the null genotype.

### Statistical analysis

Statistical analysis was performed using the SPSS 13.0 statistical software program (SPSS Inc., Chicago, IL). The χ^2^, logistic regression test, and Fisher’s exact test were applied for statistical analysis. A p<0.05 was considered to be statistically significant.

## Results

### BaP 7,8-diol 9,10-epoxide (BPDE)-like DNA adduct detected in pterygium

**Figure 1:**
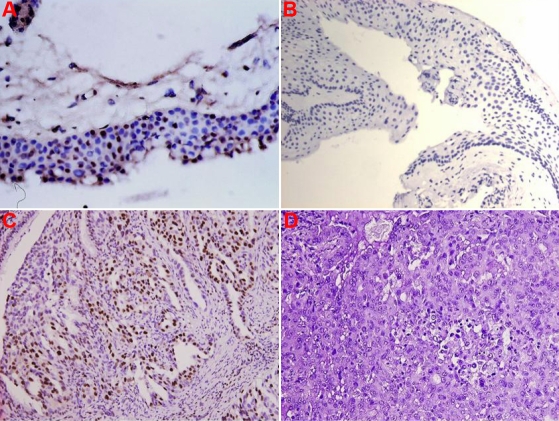
Representative positive and negative immunostaining for BaP 7,8-diol 9,10-epoxide (BPDE)like DNA adducts in paraffin sections of pterygium epithelial cells. Representative positive immunostaining (brown signal) in the epithelial layer is shown in **A** (200×), negative immunostaining is shown in **B** (200×), a lung cancer section with high BPDE-like DNA adduct levels used as the positive control is shown in **C** (brown; 200×), and a lung cancer section with no detectable BPDE-like DNA adduct levels, which was used as the negative control, is shown in **D** (200×).

In the pterygium group, 69 (67.0%) pterygial specimens scored 0, eight (7.8%) were +, 12 (11.6%) were ++, and 14 (13.6%) were +++. The detection rate of the BPDE-like DNA adduct was 25.2% if a score of 0 and + were considered to be negative for BPDE-like DNA adduct staining and ++ and +++ were considered positive (setting the cutoff level at 10%). If a score of 0 was considered to be negative and +, ++, and +++ to be positive (setting the cutoff level at 1%), the positive rate of detection was 33.0%. The BPDE-like DNA adduct staining was limited to the nuclei of the epithelial layer and subepithelial fibrovascular layers (Table 1; Figure 1). This result was similar to our previous report [33].

**Table 1:** The BPDE-like DNA adduct levels in pterygium analyzed by immunohistochemistry.

**Parameter**	**BPDE-like DNA adducts**	**%**
-	69	67.0
+	8	7.8
++	12	11.6
+++	14	13.6

The results were evaluated independently by three observers and scored for the percentage of positive nuclei: score -, no positive staining; score +, from 1% to 10%; score ++, from 11% - 50%; score +++, more than 50% positive cells. BPDE DNA adducts analyzed in this study are based on prior pterygium samples [25].

### *CYP1A1* and *GSTM1* polymorphisms in pterygium

To verify the distribution of *CYP1A1* and *GSTM1* polymorphisms in pterygium, the polymorphisms of *CYP1A1* and *GSTM1* in pterygium and control groups were analyzed by PCR-RFLP (restriction fragment length polymorphism) and PCR. The results for the genotypes of *CYP1A1* and *GSTM1* in pterygium are shown in Table 2. The analysis of the *CYP1A1* MSPI polymorphisms in pterygium showed that 34 (33.0%) were homozygous for the m1/m1 genotype, 15 (14.6%) were homozygous for the m2/m2 genotype, and 54 (52.4%) were heterozygous for the m1/m2 genotype. The analysis of the presence of *GSTM1* polymorphisms or the null type in pterygium showed that 60 (58.3%) were the present type and 43 (41.7%) were the null type.

**Table 2:** *CYP1A1* and *GSTM1* polymorphisms in pterygium analyzed by PCR-RFLP and PCR.

**Gene**	**Number**	**%**
*CYP1A1*		
A (m1/m1)	34	33.0
B (m1/m2)	54	52.4
C (m2/m2)	15	14.6
*GSTM1*		
Null	43	41.7
Present	60	58.3

### Correlation of BaP 7,8-diol 9,10-epoxide (BPDE)-like DNA adduct levels and *CYP1A1* and *GSTM1* polymorphisms in pterygium

Previous reports have indicated that *CYP1A1* and *GSTM1* polymorphisms correlated with BPDE-like DNA adduct formation [34,35]. To verify this point, the relationships between BPDE-like DNA adduct levels and *CYP1A1* and *GSTM1* polymorphisms in pterygium were analyzed. As shown in Table 3, only the *CYP1A1* polymorphisms correlated with BPDE-like DNA adduct levels. The BPDE-like DNA adduct in patients with the m2/m2 polymorphism was higher than in the m1/m1 and m1/m2 groups (p=0.006). Additionally, there was no correlation between the *GSTM1* polymorphism and the BPDE-like DNA adduct in this study group.

**Table 3:** Relationship of BPDE-like DNA adduct levels and *CYPA1* and *GSTM1* polymorphisms in pterygium patients.

** **	**BPDE-like DNA adduct levels**		** **
**Gene**	**Negative**	**Positive**	**p value**
*CYP1A1*			
A	29	5	** **
B	29	25	** **
C	11	4	0.006
*GSTM1*			
Null	17	17	** **
Present	25	44	0.205

The results were evaluated independently by three observers and scored for the percentage of positive nuclei: negative, 0%; positive, more than 11% positive cells. BPDE DNA adducts analyzed in this study are based on prior pterygium samples [25].

### *CYP1A1* polymorphism is a risk factor of BaP 7,8-diol 9,10-epoxide (BPDE)-like DNA adduct formation

The influences of *CYP1A1* and *GSTM1* polymorphisms and gender in BPDE-like DNA adduct formation were calculated by logistic regression analysis. Among the characteristics, only the *CYP1A1* polymorphisms were significant risk factors (Table 4; p=0.001, 95% Confidence Interval 2.451–38.185). The risk of BPDE-like DNA adduct formation for patients with *CYP1A1* m2/m2 and m1/m2 was 9.675 fold more than that of patients with m1/m1 types. This suggests that *CYP1A1* polymorphisms are significant as risk factors in BPDE-like DNA adduct formation in pterygium patients.

**Table 4:** The effects of gender, *CYP1A1*, and *GSTM1* polymorphisms on DNA adduct levels in pterygium patients.

**Parameters**	**OR**	**Unfavorable/favorable**	**95%CI**	**p**
Gender	0.343	Female/Male	0.082–1.432	0.142
*CYP1A1*	9.675	A/B+C	2.451–38.185	0.001
*GSTM1*	0.453	Null/Present	0.159–1.292	0.139

## Discussion

To our knowledge, this is the first study to analyze the correlation of genetic polymorphism and BPDE-like DNA adduct formation in pterygium. Previous studies have shown that DNA adduct levels are associated with *CYP1A1* and *GSTM1* polymorphisms [34-39]. However, in our study we found that only the *CYP1A1* polymorphisms were associated with BPDE-DNA adduct formation in pterygium. If genetic polymorphisms are not associated with individual susceptibility to carcinogenic PAHs, DNA repair capacity may play an important role in the susceptibility to DNA damage. Previous reports support the idea that the capacity of BPDE-DNA adducts to be removed from peripheral lymphocytes after exposure to BPDE in vitro, measured by ^32^P-postlabeling and host-cell reactive assay, is significantly lower in cancer patients compared to healthy persons [40-42]. Common polymorphisms in DNA repair enzymes have been hypothesized to result in reduced capability to repair DNA damage [43,44]. Several reports have indicated that polymorphisms of DNA repair genes are associated with pterygium formation [45,46]. Thus, reduced DNA repair capacity appears to contribute to DNA adduct formation in pterygium.

GST is one of the antioxidant defense enzymes that contributes to the protection against reactive oxygen species [47,48]. The *GSTM1*-null type was reported to be associated with cutaneous photosensitivity [49,50], so *GSTM1* null may be associated with the photosensitivity of corneal limbal cells. Our previous report indicated that lack of *GSTM1* (*GSTM1*-null type) contributes to susceptibility of pterygium formation in early onset pterygium but is not associated with late onset pterygium [46]. In the present study we did not find an association between the *GSTM1* polymorphism and BPDE DNA adduct levels. Therefore, we suggest that the role of *GSTM1* in pterygium formation is more important in antioxidant defense than in PAH metabolism.

PAH compounds are the products of incomplete combustion of organic material and are thus ubiquitous in the environment (IARC, 1983). Occupational exposure to PAH compounds increases the risk of lung and, putatively, other cancers and is highest in coke oven workers, other workers in the steel industry, asphalt and bitumen workers, and those exposed to gasoline exhaust and working with gasoline. The best known carcinogen in cigarette smoke, BaP, has been experimentally shown to induce G:C–T:A transversions [51], which are the main mutation types in smoking-related lung cancer [52]. Our present study shows that BPDE-like DNA adduct levels correlated with a *CYP1A1* polymorphism. The mutation hotspots of *p53* in human lung tumors (codons 154, 157, 158, 245, 248, and 273) are caused by the BPDE-N2-dG adduct [11]. Thus, an evaluation of DNA adducts induced by BaP and other PAHs is suitable as a risk marker of *p53* mutation. The *p53* tumor suppressor gene is one of the most commonly mutated genes observed in human tumors. The mutation of *p53* has been noted in more than 50% of all human cancers [53-55].

Additionally, our previous study showed that BPDE-like DNA adducts are indeed detected in pterygium samples and are minor contributors to the abnormal *p53* gene [33]. Therefore, we hypothesize that after exposure to environmental PAHs, the *CYP1A1* polymorphism may result in high levels of BPDE-like DNA adduct formation contributing to *p53* or other tumor suppressor gene mutations to induce pterygium formation.

Our previous study detected BPDE-like DNA adducts in pterygium paraffin sections [33]. In the present study, we also found that the *CYP1A1* polymorphism correlated with BPDE-like DNA adduct formation in pterygium. These findings seem to provide molecular evidence to support the idea that not only ultraviolet radiation but also environmental exposure is involved in pterygium pathogenesis.
